# Meeting the Behavioral Health Needs of Health Care Workers During COVID-19 by Leveraging Chatbot Technology: Development and Usability Study

**DOI:** 10.2196/40635

**Published:** 2023-06-08

**Authors:** Maga Jackson-Triche, Don Vetal, Eva-Marie Turner, Priya Dahiya, Christina Mangurian

**Affiliations:** 1 Department of Psychiatry and Behavioral Sciences University of California, San Francisco San Francisco, CA United States; 2 Amwell Boston, MA United States; 3 UCSF Health University of California, San Francisco San Francisco, CA United States

**Keywords:** chatbot technology, health care workers, mental health equity, COVID-19, mental health chatbot, behavioral health treatment, mental health screening, telehealth, psychoeducation, employee support

## Abstract

**Background:**

During the COVID-19 pandemic, health care systems were faced with the urgent need to implement strategies to address the behavioral health needs of health care workers. A primary concern of any large health care system is developing an easy-to-access, streamlined system of triage and support despite limited behavioral health resources.

**Objective:**

This study provides a detailed description of the design and implementation of a chatbot program designed to triage and facilitate access to behavioral health assessment and treatment for the workforce of a large academic medical center. The University of California, San Francisco (UCSF) Faculty, Staff, and Trainee Coping and Resiliency Program (UCSF Cope) aimed to provide timely access to a live telehealth navigator for triage and live telehealth assessment and treatment, curated web-based self-management tools, and nontreatment support groups for those experiencing stress related to their unique roles.

**Methods:**

In a public-private partnership, the UCSF Cope team built a chatbot to triage employees based on behavioral health needs. The chatbot is an algorithm-based, automated, and interactive artificial intelligence conversational tool that uses natural language understanding to engage users by presenting a series of questions with simple multiple-choice answers. The goal of each chatbot session was to guide users to services that were appropriate for their needs. Designers developed a chatbot data dashboard to identify and follow trends directly through the chatbot. Regarding other program elements, website user data were collected monthly and participant satisfaction was gathered for each nontreatment support group.

**Results:**

The UCSF Cope chatbot was rapidly developed and launched on April 20, 2020. As of May 31, 2022, a total of 10.88% (3785/34,790) of employees accessed the technology. Among those reporting any form of psychological distress, 39.7% (708/1783) of employees requested in-person services, including those who had an existing provider. UCSF employees responded positively to all program elements. As of May 31, 2022, the UCSF Cope website had 615,334 unique users, with 66,585 unique views of webinars and 601,471 unique views of video shorts. All units across UCSF were reached by UCSF Cope staff for special interventions, with >40 units requesting these services. Town halls were particularly well received, with >80% of attendees reporting the experience as helpful.

**Conclusions:**

UCSF Cope used chatbot technology to incorporate individualized behavioral health triage, assessment, treatment, and general emotional support for an entire employee base (N=34,790). This level of triage for a population of this size would not have been possible without the use of chatbot technology. The UCSF Cope model has the potential to be scaled, adapted, and implemented across both academically and nonacademically affiliated medical settings.

## Introduction

### Background

There is growing evidence of a substantial short-term—and possible long-term—adverse impact of the COVID-19 pandemic on the mental health and emotional well-being of health care workers [1]. “Hidden” frontline (support staff such as custodial, dietary staff, ancillary staff, etc) and nonfrontline health care workers all faced rapid, sometimes dramatic, changes in the structure of health care delivery and, therefore, their day-to-day work. One dramatic change was the large-scale transition from in-person care to video telehealth assessment and treatment. A rapid review and meta-analysis examining the psychological effects of emerging virus outbreaks on health care workers found evidence of high psychological morbidity and persistence of symptoms [2]. Evidence from the studies of COVID-19 health care workers across the world reveals consistent findings of significant emotional distress including symptoms of anxiety, depression, insomnia, and constellations of symptoms consistent with posttraumatic stress [3-6]. In fact, the mental health research output in response to the COVID-19 pandemic far outnumbered prior outbreaks (eg, Ebola and H1N1).

Early in the pandemic, there was speculation from anecdotal reports that suggest that the pandemic might heighten the risk of self-harm because of increased financial strain, suspected increase in rates of domestic violence, quarantine with associated social isolation, and increased rates of substance use and addiction. The *New York Times* reported the suicide of a New York City–based emergency room physician thought to be related to her experience working at the COVID-19 frontline [7]. These concerns emphasized the urgent need for the development and implementation of prevention, triage, assessment, and treatment strategies [8].

Reports of isolation and loneliness indicated that quarantine itself could have an adverse psychological impact [9]. A rapid review found an adverse impact of quarantine on sleep, mood, anxiety, irritability, and general frustration [10]. Nonfrontline health care workers were exposed to significant stress related to rapid changes in health care delivery models such as the implementation of at-a-distance and additional telehealth or video procedures, protocols, and tools to maintain access for ambulatory patients [11,12]. With the addition of shelter-in-place orders, school closings, and other directives and guidelines designed to protect against and mitigate the spread of COVID-19, there was consensus that all health care workers were experiencing unparalleled exposure to stress-inducing circumstances and events that required emotional support [13-17].

Given the stress of the COVID-19 pandemic on the general population, “mental health chatbots” have emerged and have been implemented across multiple settings to reduce the mental health burden of the pandemic [18,19]. Chatbots have been shown to be an effective tool for mental health triage and assessment in workplace settings, allowing for employee anonymity while still providing access to needed mental health support and care [20-22]. Chatbot technology has the ability to provide large-scale triage as well as target-specific user populations. In addition, chatbot conversational agents are able to provide evidence-based strategies to improve mental health and well-being, including cognitive behavioral therapy and mindfulness training [23]. Mental health chatbot programs have been successful in improving psychological well-being and lessening symptoms of anxiety, depression, and perceived stress, and chatbots can offer an engaging, user-friendly option to manage mental health needs [24-30]. With the traditional in-person, face-to-face approach to providing mental health interventions being less feasible during the pandemic, mental health chatbots and other digital services are reliable tools to improve access to mental health screening, treatment, and psychoeducation [31,32].

### Objective

At the University of California, San Francisco (UCSF), to respond to employee concerns about access to COVID-19 crisis emotional support services, the Department of Psychiatry and Behavioral Sciences (DPBS) partnered with the Human Resources Department and the Digital Health Innovation Service to design, develop, and implement a program to provide triage and access to care for all employees, regardless of the discipline or role, across the health care system by leveraging chatbot technology [33]. The UCSF Faculty, Staff, and Trainee Coping and Resiliency Program (UCSF Cope) was designed in the spring of 2020 to not only address COVID-19–related stress and anxiety, including moderate to severe symptoms of distress, but also acute onset, or exacerbation, of behavioral health disorders. Unlike programs that focused only on the behavioral needs of frontline physicians and nurses, the UCSF Cope was designed to target all employees, including custodial support and ancillary staff. Given the recent release of the National Plan for Health Workforce Well-being by the National Academies of Medicine [34], we provide details from our experience of building this novel program in the hope that it provides others with ideas as they try to promote the well-being of their own employees. In this manuscript, we described the overall program (conceptualization, approach, and funding); details about all 4 program components (chatbot triage or screening, individual treatment, website, and special interventions); and data collection and evaluation.

## Methods

### Overall Conceptualization, Approach, and Funding

The specific intent of the UCSF Cope was to provide (1) easy, real-time access to a wide range of curated, up-to-date, web-based self-management tools (eg, apps, videos, and webinars); (2) timely access via telehealth to in-person triage, assessment, and treatment for those requiring urgent evaluation; and (3) group-level interventions, including treatment support groups and nontreatment gatherings for groups experiencing particular stress related to their unique roles, circumstances, and experiences. The target population comprised all UCSF employees (faculty, house staff, trainees, and staff; N=34,790).

Using a population health approach, program designers determined that those needing services would likely fall into 3 broad categories of symptoms, namely, mild, moderate, and severe, with the need to distinguish and address those requiring urgent or emergency intervention. The first category included those experiencing mild to moderate distress related to overall uncertainty in a setting of rapid, radical changes in the structure and experience of work, home, and community. This included stay-at-home orders, school closures, changes in work practices and procedures, uncertainty in reliable access to personal protective equipment, and accompanying fears about personal risk. This category could loosely be described as the “worried,” but essentially, “well,” meaning that additional emotional support could take the form of a wide array of web-based self-management tools, with ongoing access to additional behavioral health triage and assessment if, and when, needed. The second category included those experiencing moderate to severe symptoms of anxiety and distress, signaling the possibility of new onset of a behavioral health disorder requiring specific triage, assessment, and treatment. The third category included those with an existing behavioral health disorder experiencing worsening symptoms and possibly signaling exacerbation, relapse, or the onset of a new illness requiring reevaluation of treatment. Those endorsing acute, urgent, and emergency symptoms such as thoughts of self-harm, thoughts of harming others, a fear of violence (self and other), and child endangerment were immediately referred to emergency care resources. All the groups had unlimited access to UCSF Cope–specific web-based materials. The UCSF Cope was funded through various sources including UCSF Health, philanthropy, and in-kind resources. UCSF partnered with the Conversa Company to help develop the digital platform for the chatbot technology, which was managed by the UCSF Center for Digital Health Innovation.

### Development and Implementation of Program Components

#### Mental Health Chatbot Triage and Screening

The chatbot is an algorithm-based, automated, interactive artificial intelligence (AI) conversational tool that uses natural language understanding to engage users by presenting a series of written questions and simple multiple-choice answers. The goal of each chatbot session is to guide users to services appropriate for their needs [35]. Chatbots are panel-based systems, meaning that user answers determine which “panels” of questions and information are presented next. The planned net effect is for users to be directed to content relevant to their particular circumstances.

Initial entry into UCSF Cope services was achieved through a Conversa Digital Chatbot Screener using questions following an algorithm assessing a hierarchy of symptoms, with initial queries about urgency. In addition to the chatbot technological expertise, clinicians with expertise in the management of mental illness worked to create a broad set of mental health screening questions. The queries included a wide range of symptoms (Textbox 1). Screening questions emphasized sensitivity rather than specificity because the critical issue was ensuring that no employee in need of mental health services was missed. User responses directed employees to resources.

Immediately before launch, programmers and volunteers beta-tested and refined the chatbot technology to ensure ease of use and accuracy in both the capture of responses and the functioning of algorithm pathways. Notably, designers felt it was important to have the triage and assessment option as a default for the chatbot screener. Therefore, inconsistent responses defaulted to higher-intensity assessments and treatment options.

**Figure 1:**
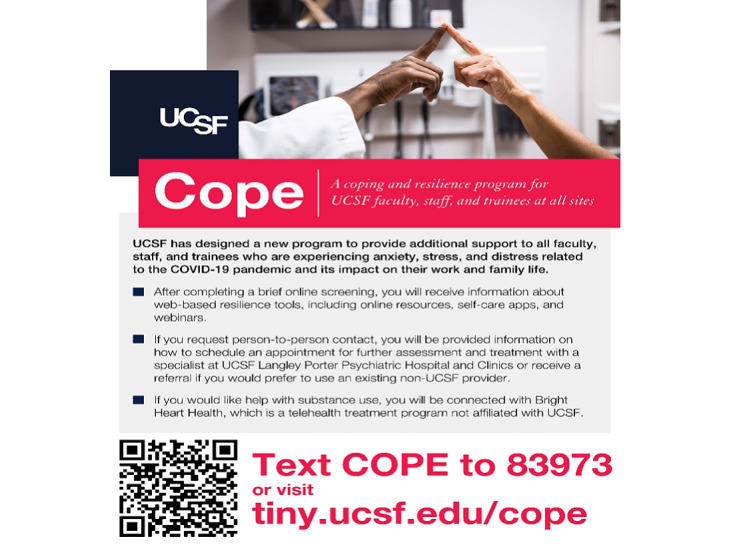
University of California, San Francisco Faculty, Staff, and Trainee Coping and Resiliency Program (UCSF Cope) outreach flyer. UCSF: University of California, San Francisco.

Users could access the chatbot screener by 1 of 3 ways: texting “Cope” to a number (83973); scanning a poster or web-based QR code (this code was also placed on all computer screensavers); or via web link [36] (Figure 1; Multimedia Appendix 1 includes a screenshot of the Cope chatbot mobile interface).

The program was advertised widely through the following methods: (1) added to screen savers at all computer stations in hospital settings and hub rooms; (2) added to the Carelinks website, a primary hub of medical center traffic on the intranet; (3) included in all manager weekly emails; (4) mentioned on all COVID-19 town halls; (5) placed on the UCSF coronavirus web page; and (6) mentioned by managers who were encouraged to do so at all staff or manager meetings. Staff without access to technology were provided with a telephone number to connect them to services.

**Figure 2:**
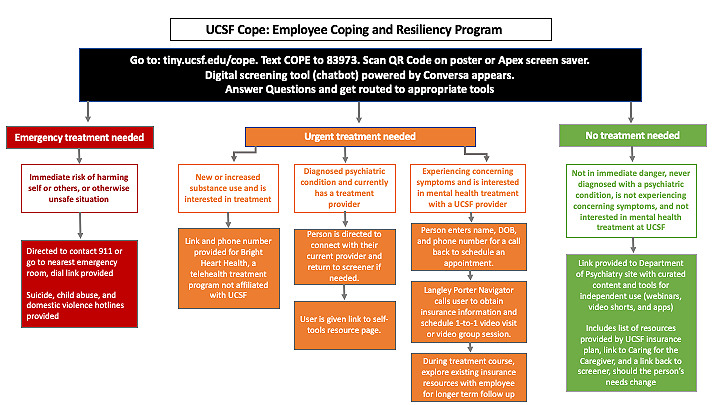
University of California, San Francisco Faculty, Staff, and Trainee Coping and Resiliency Program (UCSF Cope) triage based on behavioral health need. DOB: date of birth; UCSF: University of California, San Francisco.

A unique aspect of the UCSF Cope chatbot was the ability to triage employees to services tailored to their needs based on their individual responses (Figure 2). Those endorsing the immediate risk of self-harm, harm to others, or unsafe circumstances were directed to call “911” or to go to the nearest emergency room via a dial link to find the telephone number and address of that emergency room. The chatbot also provided suicide, child endangerment, and domestic violence hotline contact information. Those endorsing new or increased substance use and seeking treatment were given a link and telephone number to a 24-7 telehealth addiction treatment program contracted by UCSF Health [37]. Those experiencing moderate to severe symptoms were directed to triage and assessment services and were asked to leave contact information for a callback from an in-person navigator. The navigators were UCSF DPBS staff paraprofessionals. Multimedia Appendix 2 provides a detailed overview of the chatbot screening process.

**University of California, San Francisco (UCSF) Faculty, Staff, and Trainee Coping and Resiliency Program chatbot questions to triage employees to the appropriate level of care.**

**Questions (yes or no responses)**

- Are you a current UCSF faculty, staff, or trainee?
- Are you over 18 years old?
- Are you having thoughts of suicide or self-harm, or any thoughts to hurt or kill others?
- Are you or a child in a situation that is currently unsafe?
- Is your primary concern related to an increase in or problem use of alcohol or other substances?
- Have you been feeling any of the following?
  Overwhelmed, hopeless, angry, irritable, or lonely
  An increase in sadness, anxiety, or worry
  New of increased sense of fear or panic
  Experiencing stress related to COVID-19 issues?
- Are you experiencing any of the following?
  Social withdrawal
  More difficulties falling or staying asleep
  Increased struggles with work, school, or home duties
  Increased trouble with concentration or decision making
  More trouble pushing undesired throughs out of your mind
  An increase in disturbing thoughts
- Have you been diagnosed with a psychiatric condition in the past?
  - (If yes): Are you currently being treated for this condition?
- Do you have a mental health provider (psychologist, therapist, psychiatrist, counselor)?
  - (If yes): Are you able to see this person for treatment at this time?
  - (If no): Would you like to receive clinical services with a mental health provider through UCSF?
- Do you have any other issues you would like to discuss with someone?
- Would you like to be directed to specific web-based well-being resources?

#### Individual Treatment

**Figure 3:**
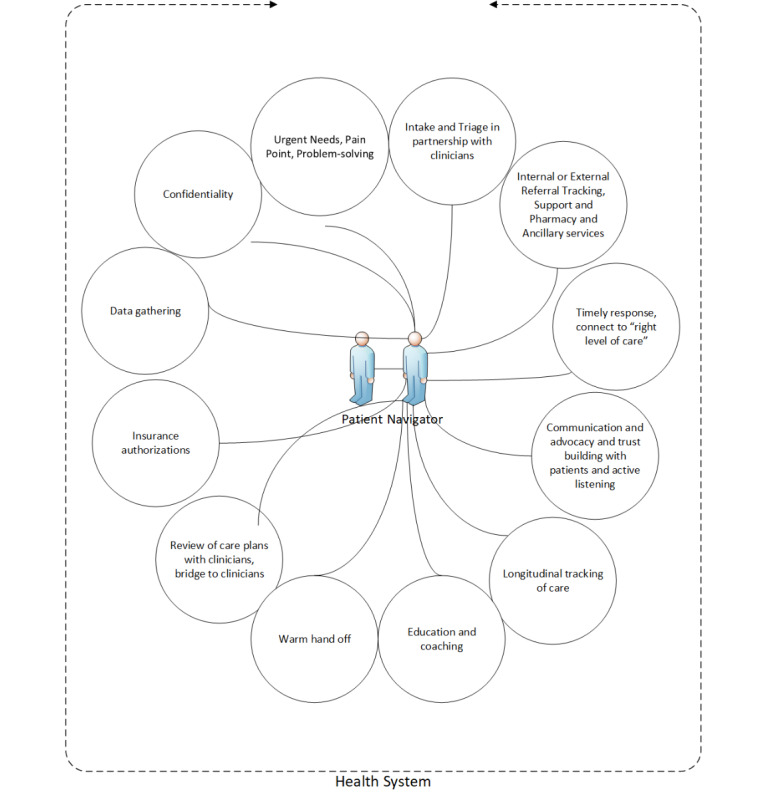
University of California, San Francisco Faculty, Staff, and Trainee Coping and Resiliency Program’s navigator role.

The in-person navigator received contact information for all employees with moderate to severe symptoms who elected that choice in the chatbot. The goal for the in-person navigator callback was within 48 hours. This dedicated navigator, who worked in the UCSF DPBS, contacted the employee directly (Figure 3) for further triage of the employee’s behavioral health needs. The navigator was able to facilitate priority access for an intake video telehealth visit with a clinician within 14 days. Treatment service options included medication assessment and management, individual psychotherapy, and group psychotherapy. We reviewed the number of referrals to navigators and projected referrals to determine the number of clinics and physicians needed. Philanthropy reimbursed clinicians for their time to provide care via special employee clinics.

Patient navigators have been used successfully in other contexts in psychiatry and primary care [38]. In general, the patient navigator is a nonlicensed employee who functions as an extension of the clinical team and works with patients to assess and overcome barriers to health care access. For UCSF Cope, navigator tasks included intake, insurance coverage review, support for referrals for specialty care, and the coordination of appointments related to the treatment of the primary behavioral health condition. The qualifications and skills of a successful navigator include 2 to 3 years of health care experience; attention to detail; compassion; excellent listening skills; the ability to follow through; and a thorough understanding of health care facilities, policies, and procedures (Figure 3).

For employees triaged to the navigator, the navigators determined whether to refer the patient to (1) emergency services, (2) their existing provider, or (3) a UCSF Cope treatment provider or to provide the patient with self-care resources, advice, and instructions on how to reconnect in the future as needed. For employees triaged for individual treatment, the navigator scheduled an appointment at the UCSF Cope clinic. Insurance was billed whenever possible; however, early during the pandemic, the program also provided care to employees without insurance.

The goal of the UCSF Cope clinic was to provide outpatient mental health services to stabilize employees and then transition these individuals to other UCSF programs or outside providers. Staff mental health clinicians (psychiatrists, psychologists, social workers, nurse practitioners, and therapists) provided care in clinics established exclusively for UCSF employees. A minimum of 2 providers were available for multiple half-day sessions 5 days a week with the flexibility of having early morning, midday, and end-of-day appointments to accommodate employees’ schedules and to minimize the risk of employees not seeking care because of competing work demands. The clinicians provided medication management and psychotherapy (eg, cognitive behavioral therapy, motivational interviewing, and dialectical behavioral therapy) as clinically indicated via Zoom (Zoom Video Communications Inc). The duration of initial visit is 60 minutes and that of follow-up visits is 30 to 45 minutes, with the frequency determined by the treating clinician. Each patient also participates in one 90-minute dialectical behavioral therapy or crisis management skills group per week. At the end of treatment, they are transferred to other UCSF programs or outside programs for ongoing mental health treatment as long as clinically indicated.

#### Website

In partnership with other academic institutions, such as the University of California, Los Angeles; Stanford University; and Columbia University, UCSF DPBS faculty and staff developed evidence-based content (web-based content, video shorts, webinars, and curated list of mental health apps) that was rapidly uploaded to this publicly available website. Many videos were intentionally short and targeted to deliver evidence-based stills. Given the evidence that managers play a key role in promoting workforce well-being during times of stress, website developers created specific web-based resources and videos to help managers provide support to their employees [39].

All employees, including nonchatbot users, had access to the DPBS website. Chatbot users reporting mild to moderate distress, but not endorsing symptoms consistent with onset, or the exacerbation of a behavioral health disorder were specifically directed to the website via the UCSF Cope chatbot [40]. As the pandemic evolved, website developers provided content tailored to the special needs of the UCSF community. Examples include content on the emotional impact of San Francisco Bay Area fires and fire risk; civil or racial and ethnic unrest and demonstrations; pandemic fatigue; identifying and addressing high-risk behaviors of teens and young adults; and an expanded family section with age-specific content for working parents managing remote student learning. Realizing the disproportionate impact of the pandemic on low-income populations, including those with limited English proficiency, we noted the need to translate key content and videos of the publicly facing website into the 6 threshold languages in the San Francisco Bay Area (Cantonese, Mandarin, Russian, Spanish, Tagalog, and Vietnamese) [41,42]. Some content was translated into Spanish; however, translating it into the full complement of languages was a work in progress.

#### Special Interventions

The UCSF Cope provided direct outreach to determine the need for unit-level tailored interventions. This included town halls, resilience groups, and wellness workshops. Outreach included departments and divisions across the university’s 4 major schools (medicine, nursing, pharmacy, and dentistry) and specific inpatient and outpatient units in addition to emergency services. For these specific interventions, UCSF prioritized the highest-risk frontline providers (eg, intensive care unit and emergency department) and “hidden” frontline workers (eg, custodial staff and nutrition services) for specific and targeted outreach [2,43]. We also created a special web-based link for special interventions. Given the large volume of outreach and coordination tasks, UCSF Cope designated a program manager and an administrative assistant to manage the workflow. The program manager set up a prioritized universal tracking sheet to manage department or unit outreach activities across the sites. In addition, to facilitate engagement and help build trust, we engaged departmental or division champions to both assess and clarify particular needs and participate in any department or division-specific town halls.

UCSF Cope used evidence-based strategies to promote resilience (eg, mindfulness, gratefulness practice, and mental health stigma) [44,45]. As pandemic conditions evolved, program coordinators modified materials to address stressors experienced by employees who were most at risk for mental health sequelae [46]. In addition to frontline workers, this included employees belonging to the following high-risk groups: (1) women (especially those with increased childcare responsibilities because of school and day care closures); (2) early career clinicians and faculty (many of whom had to develop web-based materials and provide emotional support for stressed students); (3) those experiencing financial stress; (4) caregivers for dependent adults and family members; and (5) those at increased risk of contracting COVID-19 [47,48].

### Data Collection and Analysis Considerations

Designers developed a chatbot data dashboard to identify and follow trends in use. Data for this analysis were acquired directly through the chatbot. Users confirmed their employee status. To mitigate concerns voiced by staff regarding anonymity of data, specifically worry about being identified as having a behavioral health disorder in their workplace setting, and address the concern that fear of disclosure would prevent staff from using the chatbot, the program did not capture or report identifiable data, including the department; role in the university (faculty, trainee, staff); or demographic information. At the point in the chat where users were referred to the navigator for additional triage and assessment, users were transferred to a confidential Health Insurance Portability and Accountability Act–compliant process documented only in the medical center’s electronic medical record system.

An analysis of the data first looked at which web-based resources resonated with the population to understand how chatbot services might be more effective by targeting a particular set of patients. For example, the program might be more effective per patient if we focused primarily on patients who we knew were not currently engaged with a provider. We also wanted to understand whether there was an additional benefit of the program for those employees who had already established their own connection with services.

Website user data were collected monthly to assess the use of the written content, videos, and webinars. To assess the impact of special interventions, faculty providers completed a survey after the delivery of the intervention, which included results from a live Zoom survey during the intervention. We incorporated this evaluation component into all interventions delivered so that we could perform continuous quality improvement and tailor them to our community’s needs and feedback.

### Ethical Considerations

No ethics approval was applied for as this program development process did not meet the requirements for research needing institutional review board review [49].

## Results

### User Statistics

**Figure 4:**
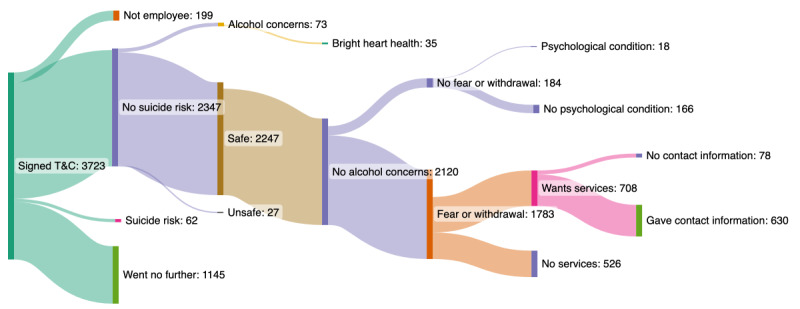
A Sankey chart illustrating at a population level; the number of employees progressing down particular engagement pathways as of May 31, 2022 (Map created with SankeyMATIC). T&C: terms and conditions.

The program began on April 20, 2020. As of May 31, 2022, a total of 10.88% (3785/34,790) of employees had accessed the technology. Engagement toward the completion of the chat is summarized in a Sankey chart in Figure 4. When the number of employees is reduced between the various states, a portion of the population exited the chat (this is equivalent to the “Went No Further” state). The Sankey chart (Figure 4) reflects the order of operations within the chat. A combination of understanding the order and rules that guide an employee to a different part of the chat and clear tracking of instances in which the employee did not answer questions provided value in our analysis. This allowed the team to build a picture of the point at which people were exiting the chat and, consequently, helped us understand which particular set of questions was helpful and not helpful in the chat.

In terms of triage, 89 individuals were referred to “911,” with 62 (70%) for thoughts of suicide and 27 (30%) reporting self or child endangerment. Among the employees reporting any form of psychological distress, 39.7% (708/1783) requested in-person services. Of the 708 employees, 630 (88.9%) provided contact information for the navigator. The session length depended on the triage responses.

Comparing the portion of the population requesting services, the data suggest that having an existing mental health provider had little, if any, impact on an employee’s interest in the individual program services when compared with those without an existing provider (43/75, 57% vs 671/1144, 58.65%, respectively; *P*=.10). Most employees with or without existing providers were also interested in web-based services, with a trend toward greater interest among those without providers (34/50, 67%, vs 148/181, 81.8%; *P*=.049). Both observations warrant further study with a larger population.

### Evaluation Outcomes

Prior published work reported on the use of individual treatments in the clinic [33]. In terms of self-help resources, as of May 31, 2022, the UCSF Cope website had 615,334 unique users, with 66,585 unique views of webinars and 601,471 unique views of video shorts.

Since the launch of UCSF Cope, the special interventions team has contacted leadership in all departments or units across UCSF sites, and >40 departments, divisions, or other units have requested and received special interventions. The town halls were particularly well received, with an average attendance of 74 attendees, and over 80% of attendees reporting that the experience was helpful.

## Discussion

### Principal Findings

UCSF Cope represents an effort to offer a user-friendly, readily accessible program that provides screening, assessment, and treatment to assist frontline and other health care workers and support staff dealing with symptoms of stress and distress related to the COVID-19 pandemic. The chatbot screening tool allowed for behavioral health triage and assessment based on employees’ responses. Notably, there were no formal claims made related to the tool. To our knowledge, this is the first pandemic-related program of its kind that targeted all health care system employees regardless of status or role and that incorporated individualized behavioral health treatment and support.

Employee engagement with the UCSF Cope chatbot screening allowed the program to identify urgent mental health needs by capturing suicidal ideation, self-injury, and self or child endangerment and to immediately refer these employees to individual treatment. Many of those referred for behavioral health triage and assessment have initiated treatment in both individual and group settings with the aid of UCSF Cope patient navigators. This program had little to no impact on existing nonemployee clinical services because these were separate clinics that were created and sponsored by philanthropy.

Health care workers experiencing mild to moderate stressors heavily used the accessible and diverse web-based self-management resources provided on the website, with >600,000 users. The special interventions catered toward specific departments and divisions were especially well received and helpful, allowing for more tailored community support to address their specific needs.

### Limitations

Because of the increasing need for mental health and emotional support for health care workers early in the COVID-19 pandemic, the UCSF Cope was rapidly developed and operationalized. The time frame from conceptualization to implementation was approximately 1 month since San Francisco’s Department of Public Health had first declared a shelter-in-place order on March 16, 2020. Therefore, designers focused on providing timely access to evidence-based care using an accessible chatbot tool but sacrificed the ability to construct a more robust data analytics plan. In addition, because of employee workplace confidentiality concerns, the chatbot did not collect demographic or role or discipline-specific information and was not able to identify trends in reuse. In an effort to encourage users to access the chatbot whenever needed, especially in the case of new onset of symptoms, users had unlimited and unrestricted access. In addition, because this program was new, designers did not have baseline data on the UCSF workforce mental health needs and thus relied on reviews of the available COVID-19 literature and provider reports. We do not have the information on those who did not use the program or the capacity to obtain user feedback.

In addition, there were three core limitations we identified in launching this UCSF Cope chatbot itself:

1. Data sparsity: one of the challenges in measuring the effect of chatbots is having enough data, even with large population sizes, to make sense of the results at the population level. This is because each chat has different paths a patient may take. This means that by the time an employee reaches the end of a chat, they are in a very small group of users who may have been served and responded to a particular question. Although the effectiveness of this chatbot and many others is fairly easy to measure as a whole, optimization of the chat is difficult owing to highly sparse data.
2. Data model interpretability: a constant feed of analyzable information must be provided to the team of specialists evolving the chat over time. This means that the data model that tracks what each employee is doing in the chat (eg, when they are exiting and when they are clicking links) needs to be easily parsed by the analytic tools. This will ensure that visualizations, as shown in Figure 4, are able to be auto-generated on demand for analysts. An overly complex data model will prohibit this. The path to success in chatbots combines clinical and behavioral domains but almost certainly will rely heavily on marketing and engagement expertise. Therefore, fast iterations of content and pathways are critical to success.
3. AI: although machine learning and AI continue to mature, there are challenges for implementation. For example, for highly directed chats that are meant to accomplish a specific goal, generally, the population is not large enough to implement a machine learning approach that optimizes some objective function (ie, chat completion).

Had we not provided user anonymity, we would have explored some additional possibilities. For example, there was a portion of the population with behavioral health symptoms that did not request an appointment. We would have spent time to design an automated extension to the patient journey for those that chose not to get an appointment but could use some ongoing additional support. As the chat was geared toward mental health crisis scenarios, another example could have been to provide an “unscheduled” session to allow a returning employee to review additional resources without having to take the whole screener again.

### Comparison With Prior Work

The UCSF Cope used the major recommendations of a recent narrative review and conceptual framework regarding clinician mental health [50]. Specifically, the program provides resilience and stress reduction training, peer and social support interventions, and normalization and provision of mental health support programs. Although there are several excellent web-based sources for promoting well-being from the National Academy of Medicine [51], Accreditation Council for Graduate Medical Education [52], Centers for Disease Control and Prevention [53], and Center for the Study of Traumatic Stress [54], UCSF Cope is unique in that it has a clear path to individual mental health treatment, if needed [51-54]. In addition, this program prioritizes, but does not limit to, clinicians on the front line. This population health approach promoted mental health equity for all employees, regardless of their role.

This program expands on prior work investigating the effectiveness of chatbot technology in mental health screening in workplace settings and facilitating web-based psychoeducation to improve mental health and well-being and the overall user-friendliness of this technology and to reduce barriers to user engagement [22,30,31]. To promote resilience, UCSF Cope incorporated a wide array of web-based, evidence-based self-management tools, to which users could be directed to via the UCSF Cope chatbot. Employees found UCSF Cope’s accessible and user-friendly web-based self-management tools to be engaging. These tools could potentially be adapted or used as models for similar programs that use web-based materials and chatbot technology. In addition, to reduce barriers to engagement, UCSF Cope materials addressed the stigma associated with mental health problems as it might prevent some employees from accessing the mental health care that they needed [55].

The UCSF Cope Program aimed to promote health equity in that every employee at the university has access to these UCSF Cope resources, regardless of their role (faculty, trainee, and staff) or financial means. Given that the COVID-19 pandemic continues to evolve and communities face additional stressors (eg, wildfires, racism pandemic, and school closures), it is critical that all employees receive the care they deserve.

### Conclusions

The UCSF Cope, with its demonstrated ability to provide large-scale triage and access to behavioral health treatment and emotional support for an entire employee base, would not have been possible without the use of chatbot technology. The ability to rapidly triage a large population and direct users to resources based on their individual responses without a chatbot or some similar technology would have required tremendous behavioral health resources not available in the current health care environment. The UCSF Cope chatbot allowed the health care system to identify employees with significant mental or emotional health needs, direct those employees to needed services, provide general resources and materials to all employees, and use existing behavioral health clinicians and staff for targeted assessment and treatment. Of importance is the fact that the UCSF Cope received a positive response from faculty, staff, and trainees experiencing the pandemic-related stressors.

This paper presents a model for large-scale behavioral health triage with an embedded, directed path to further behavioral health assessment and treatment. In this sense, it is a model that can be adapted to provide and improve general behavioral health access. Although this program was implemented under crisis conditions, it has the potential to be adapted for noncrisis conditions and for populations beyond health care workers. Given the current reports on the high levels of burnout in health care workers and the impact of burnout on satisfaction, retention, and productivity, this model may be useful in addressing the needs of highly stressed health care providers and the staff that support them [56,57].

## Appendix

Multimedia Appendix 1 Screenshot of the University of California, San Francisco Faculty, Staff, and Trainee Coping and Resiliency Program (UCSF Cope) chatbot mobile interface.

Multimedia Appendix 2 University of California, San Francisco Faculty, Staff, and Trainee Coping and Resiliency Program (UCSF Cope) chatbot process at a glance.

## Abbreviations

AI: artificial intelligence
DPBS: Department of Psychiatry and Behavioral Sciences
UCSF: University of California, San Francisco
UCSF Cope: University of California, San Francisco Faculty, Staff, and Trainee Coping and Resiliency Program

## Data Availability

Materials used for these special interventions are available on request.
