# Supplementary material for: Meeting the Behavioral Health Needs of Health Care Workers During COVID-19 by Leveraging Chatbot Technology: Development and Usability Study
Source: J Med Internet Res. 2023 Jun 8;25:e40635. doi: 10.2196/40635 (PMC10263106; doi:10.2196/40635)
Supplement: Multimedia Appendix 1 [file jmir_v25i1e40635_app1.pdf]

UCSF University of California San Francisco

Do you wish to be connected to online well being resources?

Yes

Click here for UCSF Online Mental Health Apps

Click here for UCSF Mental Health Resources

Click here for UCSF Resources on Grief and Loss

Click here for UCSF Parenting Resources

Click here for UCSF Resources for Elder Care

Click here for UCSF Resources for Climate and Wildfires
