# Supplementary material for: Meeting the Behavioral Health Needs of Health Care Workers During COVID-19 by Leveraging Chatbot Technology: Development and Usability Study
Source: J Med Internet Res. 2023 Jun 8;25:e40635. doi: 10.2196/40635 (PMC10263106; doi:10.2196/40635)
Supplement: Multimedia Appendix 2 [file jmir_v25i1e40635_app2.docx]

**Table S1.** University of California, San Francisco Faculty, Staff, and Trainee Coping and Resiliency Program (UCSF Cope) chatbot process at a glance.

| Steps | 1 | 2 | 3 | 4 | 5 | 6 | 7 | 8 |
| --- | --- | --- | --- | --- | --- | --- | --- | --- |
| Product transformation |  |  |  |  |  |  |  |  |
| Process step | - First contact | Triage | Connection with Bright Heart Health | Yellow report created | - Assign patients to APEX^a^ worklist | - Schedule appointment | - Validate benefits | - Video visit |
| Signal to start this step | - Initiated by employee | Initial screening questions completed | Affirmative answer through screening app | Cope screening platform | - Receipt of screening platform report | - Affirmation from patient over the phone that they wish to schedule | - Epic WQ^b^ 7587 | - Appointment time |
| Process step detail | - Initiate contact - Complete initial screening questions | Yellow status determined based on answers or screening questions | Provide phone number and website of Bright Heart Health | Report of individuals who screened into Yellow status generated | - Assign MRN^c^ and input demographics - Put patients in APEX worklist according to Red or Yellow status | - Call patient and get their affirmation to proceed - Get patients insurance information - Schedule appointment - Encourage MyChat sign-up | - Create MH HAR^d^ and input insurance information - Check benefits for telehealth services - Document findings | - Patient is seen - Disposition determined by patient and clinician |
| Operator (include required licensure, if any) location | - Employee | Automated | Automated | Automated | - Cope navigator | - Cope navigator | - Cope navigator | - Mental health provider |
| Location | - Web-based | Web-based | Web-based | Web-based | - Web-based | - Web-based | - Web-based | - Web-based |
| Information system | - Cope screening platform | Cope screening platform | Phone or PC | Cope screening platform | - APEX | - APEX | - Various | - Video platform |
| Equipment tools | - Phone or PC | Phone or PC | Phone or PC | PC | - PC | - Phone or PC | - Phone or PC | - Video platform, Phone or PC |
| Quality requirements | - Screening questions completed | Screening questions completed | Correct information delivered | Automated report made | - Correct categorizing of patients based on triage | - Occurs within 24 hours of triage Cope timeslot (SACC^e^ or MACC^f^ in APEX) | - Within 1-2 business days | - Documentation complete |
| Mistake proofing | - Automated process | Automated process | —^g^ | Automated process | - Quality check by second navigator | - Controlled by Epic documentation | - Controlled by Epic documentation | - Controlled by Epic documentation |
| Visual controls | — | — | — | Appearance of downloaded report in correct format | - APEX | - APEX | - Controlled by Epic documentation | — |
| Cycle time (minutes) | - 1 | 2-5 | 1 | 8 | - 5 | - 5 | - Various | - 50 |
| Operator standard work | - No | No | Yes | No | - Yes | - Yes | - Yes | - Yes |

^a^APEX: Application Express.

^b^WQ: workqueues.

^c^MRN: medical record number.

^d^MH HAR: mental health hospital account record.

^e^SACC: screening and acute care clinic.

^f^MACC: montgomery (street) acute care clinic.
^g^Not available.
